# A Case Report of a Normal Pregnancy in a Bicornuate Uterus Through In Vitro Fertilization

**DOI:** 10.7759/cureus.29152

**Published:** 2022-09-14

**Authors:** Isha Agarwal, Surekha Tayade, Sakshi Sharma

**Affiliations:** 1 Department of Obstetrics and Gynaecology, Jawaharlal Nehru Medical College, Datta Meghe Institute of Medical Sciences (Deemed to be University), Wardha, IND

**Keywords:** ultrasonography, anomalies, congenital, uterus, bicornuate

## Abstract

The case report has been done to examine the possibility of normal pregnancy achieved in the case of a rare congenital anomaly, the bicornuate uterus. A bicornuate uterus is a very rare congenital anomaly of the uterus, which falls in the class 4 category according to the classification of Mullerian duct anomalies given by the American Society of Reproductive Medicine and is associated with several obstetrics complications like malpresentation, recurrent abortions, and growth restrictions. However, to have a normal pregnancy in a bicornuate uterus, close antenatal monitoring is required, and, depending on the individual, surgical unification can be done. A 30-year-old woman with G3A2 with 34.3 weeks of gestational age with in vitro fertilization (IVF) conception came with cervical stitch in situ and oligohydramnios with liquor index 7 for safe confinement. At the time of admission, amenorrhea was present for nine months. Ultrasound at 33 weeks three days showed a single uterine live fetus weighing about 2187 grams. The interpretation of the color Doppler was also normal. Fetal heart sound was heard in the Doppler. She was operated on at 36 weeks as an emergency lower-section cesarean section procedure. The indication was that it was an IVF baby, and the female had presented with oligohydramnios on performing investigations. The patient was counseled accordingly and discharged on 22 February 2022. She was advised to come back after 15 days or SOS at the time of emergency. All the measures were suggested, including adequate rest, plenty of fluids, and a good protein diet. Most cases of the bicornuate uterus do not present with any symptoms, i.e., they are asymptomatic and can be detected during routine evaluation of the patient. However, some patients can also have symptoms like menstrual problems such as dysmenorrhea and menorrhagia. Also, along with this anomaly, associated anomalies may be present, including agenesis of the kidney and ureter. The first and foremost investigation to be done is ultrasonography, which tells about the diagnosis of the bicornuate uterus. Magnetic resonance imaging is the gold standard test for its diagnosis. However, the diagnosis in the case of asymptomatic patients is relatively tricky and requires aggressive prenatal monitoring and needs to be kept in observation to make the pregnancy successful.

## Introduction

A bicornuate uterus is one of the congenital anomalies and malformation of the uterus that is caused due to the non-fusion or impaired fusion of Mullerian ducts. Mullerian ducts, also known as paramesonephric ducts, are embryological structures important for developing the urogenital system. There are several uterus anomalies, including agenesis of the uterus, unicornuate, didelphys, septate, arcuate, bicornuate, and many more. The incidence of uterine malformations is estimated to be 3-5% in the general population [1]. The incidence of bicornuate uterus is estimated to be 0.1-0.6% [2]. A bicornuate uterus is a very rare anomaly in which the uterus is heart-shaped and not in a regular shape, that is, a pear shape. It falls in the class 4 category of Mullerian duct anomalies classification. Class 1 is the agenesis uterus, class 2 is the unicornuate uterus, class 3 is the didelphys uterus, class 4A and 4B are partial and complete bicornuate uterus, respectively, class 5 is the septate uterus, class 6 is the arcuate uterus, and class 7 consists of diethylstilbestrol-related anomalies.

A bicornuate uterus is a major cause of spontaneous abortion [3]. Based on the partition of the cervix, it is divided into bicornuate unicollis and bicornuate bicollis. Due to the abnormality, it is most likely that the reproductive outcome is not favorable in such patients. They are categorized under high-risk pregnancy and can result in preterm labor in pregnant women or recurrent pregnancy loss, which makes it challenging for the women to have a normal pregnancy. Women with recurrent pregnancy loss have a 3.2-6.9% likelihood of having a major uterine anomaly [4]. Therefore, in any case of bicornuate, the pregnancy has to be managed by taking into consideration all the obstetrical complications. The female with this anomaly may even present menstrual complaints in the reproductive age group. In a diagnosed female, antenatal monitoring should be aggressive and is of paramount importance because that would decide the fate of the current pregnancy and will favor successful pregnancy. It can also be surgically corrected by unifying the uterus, considering what the individual wants. With all the management and monitoring, it is still possible to have a normal pregnancy. One such case has been described in the following report. We have a 30-year-old female residing in Maharashtra. She was diagnosed with a bicornuate uterus but had a successful pregnancy this year.

## Case presentation

This is a case of a 30-year-old patient, Hindu by religion, and a homemaker by occupation, who came to the outpatient department (OPD) on 10 February 2022 with nine months of amenorrhea with no chief complaints. Her gestational age was 34 weeks three days with in vitro fertilization (IVF) conception along with cervical stitch in situ and oligohydramnios L1 with safe confinement. Her obstetric history suggested that the duration of marriage is 10 years in which she had conceived two times before this, but resulted in abortion. Therefore, her obstetric score was G3A2. Both conceptions were done through frozen embryo transfer. The first abortion was done nine years back when the fetus was four months and induced because of absent cardiac activity, through dilatation and curettage.

Similarly, the second abortion was also done due to absent cardiac activity at three months through dilatation and curettage. According to the maternal history, her last menstrual period was on 3 September 2021, and the calculated expected delivery date was 21 March 2022. The current period of gestation is 34 weeks three days. Her pre-menstrual history is 7-8 days every 2-3 months, irregular with scanty bleeding and clots with no dysmenorrhea.

According to the treatment history, she took Ayurvedic treatment earlier for infertility. After this, IVF treatment was taken. There is also a history of hysteroscopy done two years back, suggesting a bicornuate uterine cavity, and a history of intrauterine insemination failure three years back, ovum pickup in 2021, and cervical stitch in the third month of pregnancy. The past history is insignificant. There was no history of hospitalization. Also, there was no history of surgical or medical illness. On physical examination, she was conscious, cooperative, and oriented to time, place, and person. She had an average build. Her weight was 52 kg, and her height was 158 cm. Her body mass index was 22 kg/m^2^. She was afebrile to touch with a pulse of 90 per minute, a respiratory rate of 12 cycles per min, and a blood pressure of 120/80 mm Hg. All the systemic examinations were within normal limits. The abdominal analysis showed a 34-week uterus size in accordance with the gestation period. Cephalic presentation and fetal heart sound were present and were regular, and the fetal heart rate was 152 bpm.

On investigations, the blood group is O positive. The HIV test was negative. HBsAg was found negative. On complete blood count, hemoglobin was 11.1 gram%. A serial ultrasonogram (USG) had been done starting from 28 weeks six days, in which fetal weight was found to be 1309 grams. The ultrasound was done on 10th February, when she was 33 weeks five days pregnant, baby weight was 2158 grams, oligohydramnios was detected, and the amniotic fluid index was 7. There was a normal color Doppler flow and spectral waveform. She was prescribed medications such as third-generation cephalosporins, analgesics, and supplements like iron, calcium, protein powder, and vitamins. Her progesterone levels were also checked, and the observed value was 1.11 on 1 June 2021. Her estradiol and luteinizing hormone levels on 7 June 2021 were 85.30 pg/mL and 0.66 mIU/mL, respectively. The anti-Mullerian hormone levels were raised to 14.5 ng/mL in March 2021. On hysterolaparoscopy, cervix, endometrial cavity, and both ostia were normal, uterus was normal, anteverted, and mobile, bilateral polycystic ovaries were present, drilling was done and spillage of dye was seen on both sides. The uterus was seen bicornuate with normal ovaries and bilateral chromopertubation positive, the pouch of Douglas (POD) was clear, and there were no adhesions [5]. The semen analysis of her husband was also done to check his fertility. The interpretation was typical, with a count of 100 million per milliliter in which about 40% come under grade 4 motility.

**Figure 1:**
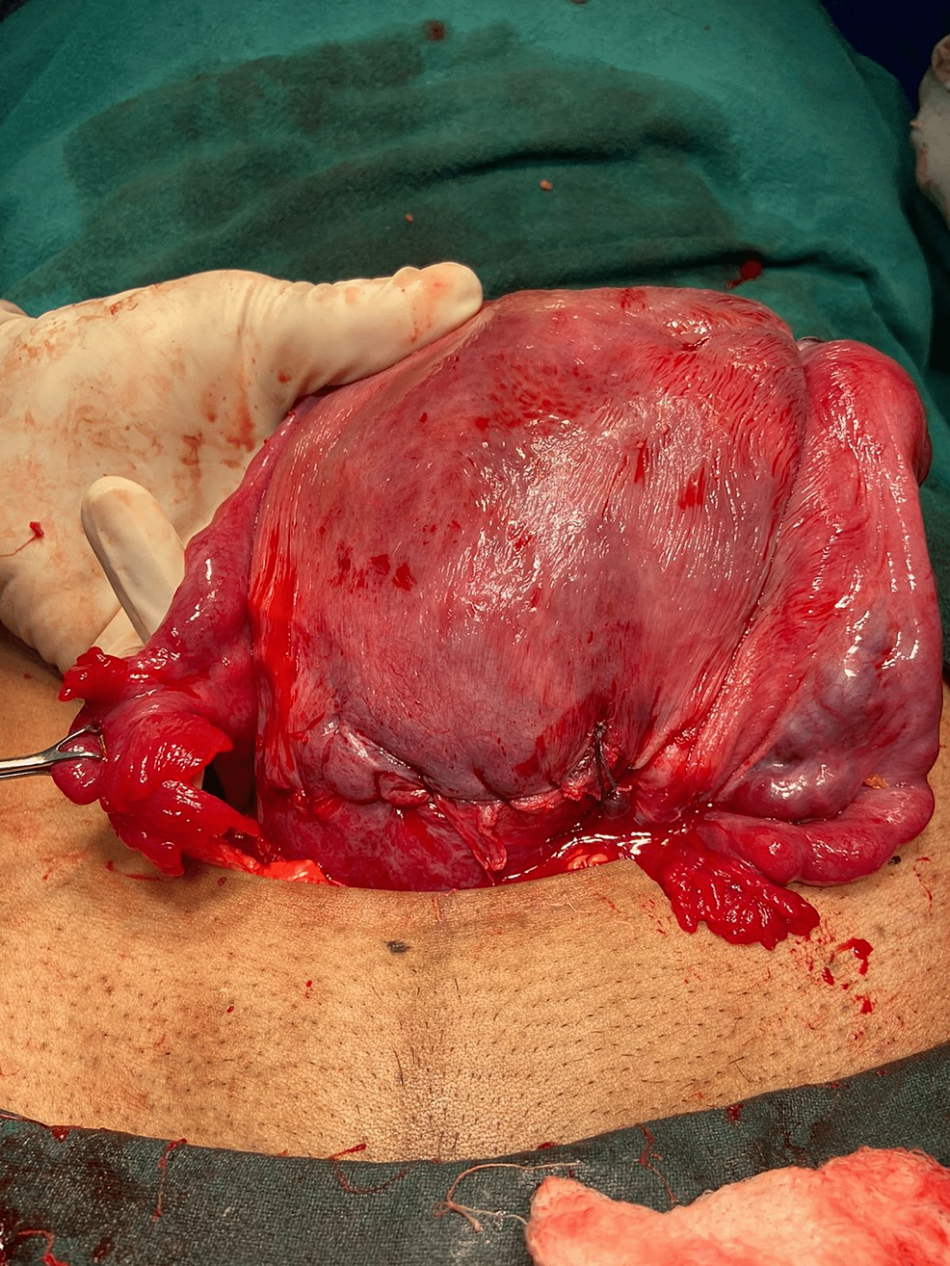
Bicornuate uterus.

Figure 1 shows a bicornuate uterus after a successful pregnancy.

Management

The process of IVF was used to conceive in this patient. On 26 March 2021, the first ovum pickup was conducted, the second ovum pickup on 17th April, the third ovum pickup on 3rd June, and the first frozen embryo transfer was done on 3 July 2021, in which three Day 5 embryos were transferred. Beta human chorionic gonadotropin was found to be positive.

The patient underwent an emergency LSCS on 16 February 2022 assisted by a second-year resident. The procedure was done under spinal anesthesia. The indication of LSCS was in view of IVF conception at 36 weeks pregnant and oligohydramnios, liquor index 7. The final diagnosis was, therefore, an emergency LSCS with presenting part vertex, female baby weighing 2.1 kg on 16th February at 11:40 AM. A Pfannenstiel incision was given. A female baby was extracted out by vertex presentation at 11:40 AM.

The baby cried immediately after birth. The cord was clamped and handed to the pediatrician. The placenta was delivered along with intact membranes and all cotyledons. The surgery was completed by closing the uterus with Vicryl number 1 with interlocking sutures. The patient was compliant enough and was shifted to the ward after the procedure. There were no intraoperative complications noted. Cervical stitches were removed during the process. The baby, on the other hand, was female, 2.1 kg weight, and the APGAR score was 8 and 9 at 1 and 5 minutes after birth, respectively. She was shifted to her relatives' side, and immunization as per schedule was given, including zero doses of oral polio, hepatitis, and BCG. Foley's catheter was removed on the third postoperative day, after which urine was passed, and checking of dressing was done on the fourth day. She was discharged on 22 February 2022. During discharge, she was advised to follow up after 15 days or SOS in case of any emergency. Also, she was asked to maintain a high-protein and -iron diet and plenty of fluids. Adequate rest was advised. Exclusive breastfeeding for six months every 2 hours or as per baby's need was advised. Complete immunization of the baby was done as per schedule. She was advised to maintain perineal hygiene and use sterile vulval pads. Medications such as tablet iron, tablet calcium, vitamin C, and protein powder were prescribed.

## Discussion

The understanding of embryology is important in congenital anomalies of the uterus. The main organ of the female reproductive system, which is the uterus, is developed from the fused caudal parts of the paramesonephric ducts forming the fundus and cervix dome. Fusion of the ducts is incomplete initially, and a septum is present in the lumina. But after some time, the septum disappears, and a single large cavity is formed. The upper part comprises the body and cervix of the uterus. But in this anomaly, there is the failure of fusion of the duct leading to the uterine defect. According to the American Fertility Society classification of Mullerian duct anomalies, the bicornuate uterus is a class IV anomaly [6].

Most of the women are, however, asymptomatic, but some may experience menstrual irregularities like dysmenorrhea. Women with an anomalous uterus have decreased chances of normal pregnancy as it does not provide a suitable physiological environment as that of the normal pear-shaped uterus. It can give rise to obstetric complications such as infertility, ectopic pregnancy, and prematurity. Therefore, the chances of conceiving through the natural method in congenital uterine malformations are thin and this is where the idea of IVF comes into action. It has been reported that the methods of IVF and embryo transfer in females with congenital anomalies of the uterus have a good percentage of successful outcomes. However, patients should be accordingly counseled and told about the associated risks. The probability of preterm delivery and cesarean section should also be considered.

Table 1 shows the prevalence of congenital anomalies of the uterus.

**Table 1:** Prevalence of congenital anomalies of the uterus

Type of anomaly	Prevalence value (%)
Arcuate uterus	11.8
Septate uterus	0.4
Unicornuate uterus	0.4
Bicornuate uterus	0.1
T-shaped uterus	0.1

Management strategies for congenital uterine anomalies

A bicornuate uterus is caused by incomplete fusion of the Müllerian ducts [7]. With the advent of new investigation techniques like MRI and 2D ultrasonography, it is much more feasible to diagnose a malformed uterus. Management of uterine malformations is not a fixed procedure and is done in accordance with the clinical history [8]. It can range from a wait-and-see approach to performing full abdominal surgery. If the diagnosis is made during adolescence, that is, before pregnancy, the preferred treatment is undergoing surgery. The surgery is only done if the patient gives consent. The surgery of choice is hysteroplasty in symptomatic individuals. In the case of women in the reproductive age group, it is necessary to rule out differential diagnoses of malformations and recurrent abortions in order to be sure about conducting the surgery [9]. According to recent advances, Strassman's utriculoplasty operation with a transverse fundal incision for the reunification of the uterine cavity has certainly proven to improve the obstetric outcome in patients with a bicornuate uterus. But to avoid the hassle of undergoing such complex surgeries, the ideal of conceiving is to adopt assisted reproductive techniques. The key to management is to remove the septum and unify the cavity. The choice of management is made according to the measure of outcome [10].

## Conclusions

The reproductive potential of the malformed uterus is assessed, with emphasis on problems of vertical and lateral fusion. With all the relevant discussion on the possibility of normal pregnancy in the bicornuate uterus, it is safe to say that the preferred method of conception is assisted reproductive techniques. Due to the reduced gestational capacity, the reproductive outcome is impaired. Uterine malformations can have a significant effect on the outcome of pregnancies. Misdiagnosis of uterine malformations may affect the outcome in pregnancy. There is a high prevalence of abortions, as seen in the above case. The likelihood of conceiving increases after consecutive failed pregnancies. However, after correction of anomaly by metroplasty, the first and second abortions decreased subsequently. Therefore, it is advisable to undergo surgery to avoid pregnancy wastage. One yet another procedure tried is cervical cerclage, which is effective in preventing second-trimester abortions and premature delivery but has no role in the first trimester. Most of the patients with bicornuate uterus do not have any symptoms in their adolescence. Therefore, metroplasty is always preferred. In patients more than 35 years of age, who are not far away from menopause, metroplasty should be done on time to prevent wastage of reproductive years. It is a laparotomy surgical procedure and can cause adhesions causing a reduction in fertility. In conclusion, an estimate of the chances of giving birth to a live-born infant can be increased by various interventions.
